# Essential Minerals and Metabolic Adaptation of Immune Cells

**DOI:** 10.3390/nu15010123

**Published:** 2022-12-27

**Authors:** Malak Alghamdi, Janelle Gutierrez, Slavko Komarnytsky

**Affiliations:** 1 Plants for Human Health Institute, NC State University, 600 Laureate Way, Kannapolis, NC 28081, USA; 2 Department of Food, Bioprocessing, and Nutrition Sciences, North Carolina State University, 400 Dan Allen Drive, Raleigh, NC 27695, USA

**Keywords:** micronutrient, malnutrition, macrophage polarization, intestinal immunity, mucosal integrity, dietary intervention

## Abstract

Modern lifestyles deviated considerably from the ancestral routines towards major shifts in diets and increased sedentarism. The trace elements status of the human body is no longer adequately supported by micronutrient-inferior farmed meats and crop commodities produced by the existing agricultural food systems. This is particular evident in the increased obesogenic adipogenesis and low-grade inflammation that fails to resolve with time. The metabolically restrictive environment of the inflamed tissues drives activation and proliferation of transient and resident populations of immune cells in favor of pro-inflammatory phenotypes, as well as a part of the enhanced autoimmune response. As different stages of the immune activation and resolution depend on the availability of specific minerals to maintain the structural integrity of skin and mucus membranes, activation and migration of immune cells, activation of the complement system, and the release of pro-inflammatory cytokines and chemokines, this review discusses recent advances in our understanding of the contribution of select minerals in optimizing the responses of innate and adaptive immune outcomes. An abbreviated view on the absorption, transport, and delivery of minerals to the body tissues as related to metabolic adaptation is considered.

## 1. Introduction

A new lifestyle has evolved in the last 80 years, owing mostly to changes in dietary habits and a rise in sedentarism. These conditions have resulted in a dramatic increase in the prevalence of noncommunicable diseases, including chronic disorders associated with four key metabolic and physiological changes (raised blood glucose, raised LDL cholesterol, excess body weight or obesity, and high blood pressure). Centers for Disease Control and Prevention maps effectively tracked the rise obesity in the US during this time, with an estimated average gain of 15 kg of body mass, 4.5 BMI units, and 18 cm of waist circumference for an average adult [1]. Excess adiposity has thus almost become the norm, and trends in diagnosed diabetes trailed the obesity data with a 15–20 year delay owing to progressive damage from sustained hyperglycemia and impaired insulin action in the target tissues [2]. Only 6.8% of US adults had optimal cardiometabolic health in 2018 [3], a critical issue that was brought into spotlight one more time by the striking relationship of metabolic health and risk of severe COVID-19 outcomes associated with immune-mediated dysfunction that leads to development of pneumonia (15%) and severe disease (5%) in unvaccinated individuals [4]. This association between excessive adiposity and deteriorated immune phenotypes is now observed worldwide, as 50–60% of the population are routinely classified as overweight or obese, and 9–12% as diabetic, in both Europe [5], Middle East and Gulf region [6], and East Asia [7].

Although the four metabolic risk factors have very different pathophysiological signatures, inflammation and oxidative stress are the common central players in their development [8,9,10]. Metabolic states directly affect systemic markers of chronic low-grade inflammation and correlate with immune activation in tissues such as fat, liver, pancreas, and the vasculature [11]. The innate immune system (granulocytes and myeloid cells) allows for a rapid proinflammatory response to injury or infection via activation of pattern recognition receptors; however, its resolution is substantially delayed in unhealthy metabolic states. The adaptive immune system (B and T lymphocytes) in turn critically depends on the innate immune cells for antigen presentation and receptor-mediated activation, otherwise unable to effectively control for autoimmune reactions. In tissues, this process seems to be maintained by the transient tissue macrophages, as well as mature tissue macrophages derived from embryonic precursors seeded in place before birth and self-renewed [12].

Failure to maintain metabolic homeostasis results in a maladaptive metabolic state that relies on tissue-resident macrophages to propagate inflammation [13]. Expansion of adipose tissue and ectopic storage of triglycerides in liver, muscle, and pancreas is also achieved by local inflammatory reactions that allow for increased perfusion and remodeling of otherwise structurally rigid tissues [14]. This is achieved by chronic upregulation of complex signaling cascades that include vasoactive amines (histamine and serotonin), eicosanoids—lipid mediators that define pro-inflammatory (prostaglandins and thromboxanes) or anti-inflammatory (leukotrienes, lipoxins, resolvins) polarization, as well as cytokines with similar polarization effects. A core cluster of effector molecules that drives pro-inflammatory responses seems to include TNF-α, IL-1β/IL-6/IL-17, IL-18/INF-γ/MCP-1 [15], and the messengers of prolonged activation (NF-κB, COX-2, iNOS) [16]. It is currently not clear to what extent the nature of an inflammatory trigger dictates the type of the mediator induced.

This situation is further complicated by an alteration in the two-way relationship between the richness and diversity of microbiota that occupies mucosal surfaces of the gastrointestinal tract or lungs, and the underlying immune tissues [17]. One common evolutionary approach to maintain tissue integrity and healthy metabolism is sensing the effector molecules (enzymes/substrates, receptors/ligands) from sequestrated cells that normally do not overlap spatially, as seen at the surface epithelium, vascular endothelium, basement membranes (epithelial-mesenchymal connection), and plasma membranes [13]. Assembly of NALP3 inflammasome in response to leaked intracellular ATP/toxins via activation of macrophage purinoceptors [18], as well as differentiation of intestinal regulatory T cells in response to metabolites secreted by the commensal microbial community [19] indicate dual regulation of the metabolic status, and maintenance of a fine balance between immunity and tolerance in the gastrointestinal tract.

Finally, another variable in a relationship between metabolic and immune health is food. The modern agricultural food systems achieved significant advances in breeding crops with increased macronutrient profiles and energy density, as well as developed an extensive set of manufacturing and processing routines that improved affordability, shelf life, and safety of contemporary food products. This was achieved, however, at a profound loss of several important phytonutrients including dietary fiber, micronutrients (vitamins and essential minerals), and phytochemicals such as phenolic metabolites [20]. Mineral malnutrition is especially widespread but difficult to quantify. Reduced consumption of organ meats, changes in geographical origin of foods, new varieties, agroecological methods of farming and preserving soils, and widespread environmental changes are in part responsible for the observed reductions [21]. The important role of minerals as a part of healthy diet as it applies to metabolic and immune health has stimulated research into altered cellular metabolism, often driven by mitochondrial dysfunction to produce metabolic disparity, which in turn influences inflammation and energy balance. These areas are the focus of the present review.

## 2. Inflammation and Metabolic Dysfunction

Inflammation is a physiological response to adverse stimuli, which may be physical, chemical, or biological. The response normally leads to the restoration of homeostasis and apoptosis of malfunctioning or necrotic cells by macrophages. In this process, macrophages undergo activation as polarization towards two opposite states, the M1 or classical (pro-inflammatory), and the M2 or alternative (pro-resolution) phenotype [22]. In addition to the pathogen defense, M2 macrophages clear apoptotic cells and mitigate inflammatory response to IL-4, IL-10, IL-13, and TGF-β signaling [23]. If the noxious stimuli are not neutralized and removed, or if the apoptotic inflammatory cells are not cleared from the inflamed tissue, the inflammatory mechanism continues, and a condition of chronic inflammation or autoimmunity can develop with recruitment of T lymphocytes and the formation of lymphoid infiltrates in the metabolic tissues [24]. This process is especially evident in the metabolic state of morbid obesity which is characterized by constant activation of the innate immune system that leads to acute inflammation [25]. Sustaining the M2 state of tissue resident macrophages would be an interesting approach to reduce circulating inflammatory mediators and thus alleviate the metabolic disorders associated with chronic inflammation.

### 2.1. Inflammation in Obesity

Obesity is a cofounding factor of many metabolic disorders. Excessive lipids in the circulation, whether they are dietary or genetically determined, trigger hyperplasia, remodeling and hypertrophy of the adipose tissue, and result in increased fat mass as an adaptation to extra energy storage. These processes have significant inflammatory underpinnings, and inflammation is linked to all stages of metabolic alterations. Metabolic dysfunction is generally observed together with a low-grade local inflammation, deficient insulin receptor signaling, and metabolic homeostasis disruption [26]. However, the precise contribution of individual macronutrients (carbohydrates, fats, proteins) to development of the obese and pro-inflammatory metabolic states has not been established.

While there is a broad consensus that increased levels of fructose-containing carbohydrates, saturated long-chain fatty acids, and branched-chain amino acids impair metabolic health, the views on their different roles are extremely polarized. This is highlighted by a generally recognized U-shaped association between mortality risk and carbohydrate consumption, with the epidemiological data from the PURE study at one extreme [27] and the Blue Zone Diets at the other [28]. These inconsistencies arise from the inherent limitations of the single nutrient approaches, and inability to correlate findings with concurrent nutrient intakes. For example, when protein is diluted in the diet by readily digestible carbohydrates and fats in the form of processed foods, protein “leverage” results in excess calorie intake, leading to rising levels of obesity and metabolic disease [29].

On the molecular level, the processes are mediated in part by increased *de novo* lipogenesis in the liver, reduced fat oxidation in mitochondria, accumulation of toxic ceramides and diacylglycerides, and activation of mTOR that ultimately degrade the insulin receptor substrate-1 (IRS-1) substrate and lead to malfunction of insulin-sensitive tissues [30]. In a remarkable overlap, deficiencies in IRS-1 substrate drive the proinflammatory phenotypes of the target tissues [31]. Similar to metabolic mediators, inflammatory cytokines like TNF-α, IL-6, and IL-1β also impair the insulin signaling pathway leading to insulin-resistant metabolic conditions [32]. Both IL-6 and TNF-α promote hepatic production of C-reactive protein (CRP), a major nonspecific reactant for the acute inflammatory phase, that is also increased in obese subjects. This further stimulates the complement system, mediates phagocytosis, and controls inflammation in the target tissues [33].

Cytokines, endothelial adhesion molecules, and chemotactic mediators within adipose tissue originate from both adipocytes, as well as resident or transitory macrophages that infiltrate the tissue [34]. These signals also activate another molecular pathway, called inflammasome, in myeloid cells which mediates the maturation and secretion of IL-1β and IL-18 by macrophages [35]. The signaling messengers have local effects on adipocytes and other resident immune cells (e.g., neutrophils, B cells, and T cells), and circulate in the periphery, where they affect the liver and skeletal muscle. In liver, this translates to increased infiltration with resident Kupffer cells and monocyte-derived recruited hepatic macrophages [36], while skeletal muscle experiences increased pro-inflammatory M1 macrophage infiltration [37].

### 2.2. Inflammation in Diabetes

The relationship between immunity and carbohydrate metabolism is bidirectional, encompassing both inflammation role in the pathogenesis of metabolic disorders and the impact of the metabolic condition, including the inflammatory signaling, on immune cell regulation [38]. At the pathophysiological level, type 2 diabetes (T2D) is primarily characterized by peripheral insulin resistance and progressive exhaustion/destruction of insulin producing pancreatic beta cells [39]. These alterations are also associated with elevated oxidative stress, which leads to the additional dysregulation of the polyol, hexosamine, and protein kinase C (PKC) pathways, as well as a rise in the formation of advanced glycation end products (AGEs) [40]. Indeed, increased oxidative stress has been a major risk factor for the most prevalent diabetic microvascular complications, including nephropathy, retinopathy, and neuropathy at the later stages of T2D. Importantly, in diabetic patients, this consistently elevated oxidative stress condition results in low-grade pathological inflammation [40].

A number of markers of inflammation are elevated in patients with diabetes, including the leukocyte count, IL-6, TNF-α, and CRP [41]. Similar to obesity, TNF-α produces metabolic perturbation in diabetic states by inducing insulin resistance via activation of IκB kinase β (IKKβ), the c-Jun aminoterminal kinase (JNK), and inhibitory phosphorylation of IRS-1 at Ser 307 [42]. A close connection between obesity and insulin resistance is exemplified by the fact that a gradual weight loss 5–15% of the original body weight over 3–10 months is sufficient to improve β-cell function and insulin sensitivity in all key metabolically active tissues: liver, skeletal muscle, and fat [43]. It has also been long recognized that anti-inflammatory treatments attenuate insulin resistance as observed with salicylic acid [44], salicilates [45], or aspirin [46], likely via inhibition of κB in the NF-κB inflammatory pathway. Inflammasome-activated IL-1β and IL-18 are the major cytokines implicated in the development of obesity- and diabetes-related insulin resistance, and some conflicting results were reported for IL-6 and the downstream STAT pathway [47].

### 2.3. Inflammation in the Gastrointestinal Disorders

Another bidirectional interaction between gastrointestinal tissues, microbiota in the gastrointestinal lumen, and host immunity, in which inflammation is critically involved, has recently been stated to have a compounding effect on metabolic diseases [48]. The gastrointestinal tract represents a major component of the immune system that maintains immune homeostasis by supporting the integrity of the intestinal epithelial barrier and recognizing the food and microbial antigens. Disruption of the epithelial barrier occurs when a double (stomach or colon) or a single (small intestine) layer of the gastrointestinal mucus is diminished [49], and the tight junction protein complexes are misassembled to allow for increased penetration of dietary components and microbial metabolites via the paracellular transport pathway [50]. This creates a unique antigen presentation environment where under normal conditions specialized epithelial microfold (M) cells recognize luminal antigens and present them to the mononuclear phagocytes (dendritic cells and macrophages) and B cells to trigger antigen-specific secretory IgA, as well as systemic IgG production [51]. In humans, these areas are more frequently localized in the distal part of the small intestine (ileum) where microbial loads start to increase [52]. This allows for timely activation and differentiation of the effector and regulatory Th cells mostly via IL-10 and TGF-β signaling to suppress the inflammatory responses of B and T cells initiated by normal food, commensal microbes, and environmental antigens [53]. The epithelial layer also expressed a significant number of extraoral bitter taste receptors family (TAS2R, 25 members in humans) that do not support the bitter sensing in the gut, but instead provide a chemosensing environment to detect and respond to dietary and microbial chemical constituents and modify their absorption [54].

In addition to ectopic lipid accumulation and chronic inflammation in the key metabolic tissues, excessive metabolic states also promote inflammation of the gastrointestinal tissues. As different parts of the gut perform distinct functions in the digestion and absorption of nutrients, the health outcomes of the gastrointestinal inflammatory disorders are highly variable. In a normal state, duodenum supports digestion of foods with pancreatic and bile secretions, as well as iron, calcium, and magnesium absorption. The jejunum absorbs most nutrients, vitamins, and minerals. The ileum reabsorbs bile acids and fluids, and colon completes absorption of fluid and electrolytes. This functional separation is partially responsible on different manifestations associated with the gastrointestinal disorders, as celiac disease primarily affects duodenum/jejunum, the Crohn’s disease is centered in ileum and spreads to colon, and the ulcerative colitis affects primarily colon starting at the anus. For this reasons, primary mineral inadequacies in celiac patients are iron, calcium, magnesium, and to a lesser degree zinc, copper, and selenium [55]. Mineral malabsorption in Crohn patients is variable but generally includes iron, calcium, magnesium, and zinc [56]. Patients with ulcerative colitis are less susceptible to mineral deficiencies, but require larger amounts of zinc, copper, and selenium to promote wound healing [57]. The altered epithelial barrier function is present in all IBD conditions and presents as increased leak-flux of water and solutes that leads to elevated antigen presentation, tissue inflammation, and diarrhea [58].

## 3. Immune Cell Metabolism and Metabolic Reprogramming

The immune system has various type of cells in its stable state, which become active in different situation in order to respond to infection, inflammation, and changes in metabolic fluxes. These responses include multiple changes in signal transduction pathways and gene expression networks to perform the suitable functions like production of cytokines, tissue remodeling enzymes, mediators, and toxic gases, in order to be able to migrate through tissues and/or undergo cellular division and proliferation. Such changes are supported by abrupt modifications of basic metabolic processes that provide immune cells with energy and bio-precursors to correlate with required immune functions. Cellular bioenergetics therefore serves as both a sensor and fundamental effector of the immune response, and this becomes even more evident in pathological metabolic states.

### 3.1. Inflamed Tissue Is a Metabolically Restrictive Environment

Cellular metabolism involves a network of biochemical reactions that utilizes nutrients and microelements generate the energy, redox equivalents, and macromolecules specific to the cell type and function. This is typically achieved first via glycolysis in the cytosol and the subsequent mitochondrial oxidative phosphorylation in the presence of oxygen. However, rapidly proliferating cells such as tumors and activated immune cells require faster energy supplies and achieve them by lowering their metabolic efficiency and relying nearly exclusive on the faster glycolytic reactions in the cytosol. This allows them to outcompete other cells and tissues for essential nutrients and microelements critical to their survival [59]. Aerobic glycolysis and pentose phosphate pathways are the main metabolic modes of activated M1 macrophages, neutrophils (respiratory burst and chemotaxis), iNOS-expressing dendritic cells, lymphocytes (effector T cells, LPS-stimulated B lymphocytes) and natural killer cells. Alternatively activated immune cells geared towards resolution of inflammation such as M2 macrophages and the regulatory T cells rely on oxidative phosphorylation from fatty acid oxidation instead [60].

Survival of activated immune cells in the metabolically restrictive environment depends on the competitive uptake of glucose and the expression of corresponding transporters on the cell surface. For this reason, many pro-inflammatory pathologies induce at least transitory, but often long-lasting state of insulin resistance in the host tissues. This is observed in patients with sepsis [61], burn injury [62], and normal pregnancy [63].

The resulting increased availability of glucose for aerobic glycolysis provides the biosynthetic precursors essential for the synthesis of nucleotides, amino acids, and lipids of rapidly growing and proliferating cells. The metabolic shifts are supported in part by interaction of glycolytic enzymes such as GAPDH with translation of IFN-γ and IL-2 mRNA to ensure unconstrained generation of pro-inflammatory signaling once glycolysis is upregulated [64], as well as activity of the glycolytic regulators such as HIF-1α under the conditions of microenvironment hypoxia [65].

### 3.2. Metabolic Reprograming during Activation of Immune Cells

**Figure 1:**
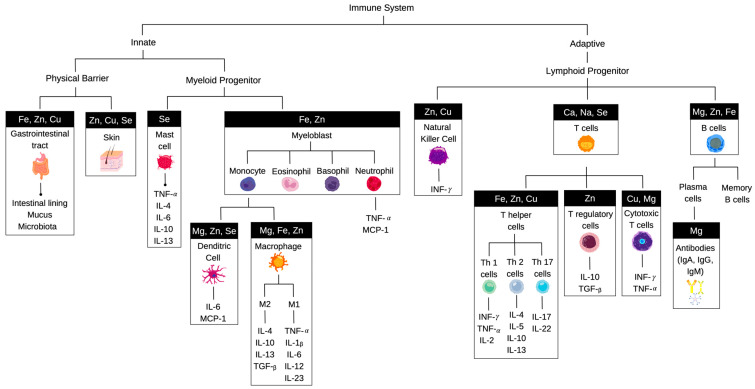
Schematic representation of the immune cell lineages of lymphoid and myeloid origin. Essential minerals that play important roles in maturation and activation of the immune cell subgroups are shown at the top. The major effector molecules produced by each subgroup include cytokines, interleukins, and chemokines as listed below the individual clades.

Active selection of metabolic pathways enables immune cells to adapt to their functional requirements, but at the same time, the metabolic state of the host directly affects the phenotype and function of immune cells. A number of minerals have been shown to be important for adequate transition between resting and activated states of the immune system, activity of the rate-limiting enzymes in respective biochemical pathways, and transcriptional factors responsible for activation of the target gene expression networks (Figure 1).

#### 3.2.1. Neutrophils

Neutrophils are a subpopulation of granulocytes (leukocytes) abundant in the blood and rare in healthy tissues. These immune cells are one of the earliest transitory responders that propagate the pro-inflammatory states via secretion of elastase 2, TNF-α and MCP-1 [47]. Neutrophils phagocytose debris, promote angiogenesis, and allow for tissue expansion and repair. They consume very low amounts of oxygen and rely primarily on aerobic glycolysis to generate ATP. A high flux through the glycolytic pathway upon activation is channeled via pentose phosphate pathway to generate NADPH and superoxide anions (oxidative burst). Neutrophils also maintain some levels of fatty acid oxidation and glutaminolysis [66].

#### 3.2.2. Mast Cells

Mast cells are another class of highly granulated tissue resident hematopoietic cells that are major effectors of IgE mediated allergies, but also disorders of the brain, gastrointestinal, and adipose tissues. They undergo early phase degranulation and late phase activation by releasing histamine, leukotrienes, TNF-α and Th2-associated cytokines such as IL-4, IL-6, IL-10, and IL-13. Both oxidative phosphorylation and glycolysis appear to be critical for rapid allergic reactions, while glycolysis is a predominant energy pathway for non-IgE activation [67].

#### 3.2.3. Macrophages

In obese metabolic states, macrophages constitute up to 40% of the stromal vascular cells in the adipose tissue [68]. Adipose tissue macrophages encompass both resident and transitory cells recruited via MCP-1, and are sometimes designated as Me due to differences in surface markers [69]. In the resting state, macrophages use energy produced from oxidative phosphorylation, and abruptly switch to glycolysis when they enter the pro-inflammatory M1 activated state (CD11b+, F4/80+ and CD11c+) via IFN-γ or LPS signaling. Their activation is dependent on the same IKK-β implicated in the pathophysiology of insulin resistance, and results in PPAR-γ mediated secretion of IL-1β, TNF-α, IL-6, IL-12, and IL-23. On the biochemical level, M1 polarization results in accumulation of succinate and malate [70] due to the Krebs cycle breaks at the level of isocitrate dehydrogenase and succinate dehydrogenase [71]. This allows for citrate shift towards fatty acid and prostaglandin synthesis, accumulation of succinate, and activation of the arginosuccinate shunt towards malate and nitric oxide production.

The alternatively activated M2 macrophages (CD206+ CD301+) participate in resolution of inflammation via secretion of IL-10, IL-4, IL-13 and TGF-β that is heavily influenced by adipose tissue eosinophils and regulatory T cells. Both IL-4 and IL-10 are two suppressive cytokines which become more abundant in the gut mucosal tissue due to exposure to helminth and microbial products derived from the gut microflora, respectively [72].

#### 3.2.4. Dendritic Cells

Dendritic cells link innate and adaptive immunity by presenting antigens to T cell receptors. They induce pro-inflammatory environment by producing IL-6 and macrophage recruitment signaling. Similar to other innate cells (dendritic cells differentiate from monocytes as do macrophages), activation of dendritic cells is critically dependent on glycolysis [73].

#### 3.2.5. NK Natural Killer Cells CD56+

Activated NK cells maintain bioenergetic profiles that are similar to Th1 cells in relying predominantly on glycolysis and glutaminolysis for energy supply. Like other innate immune cells described above, NK responses are rapid and nonspecific. The observed shifts to a higher metabolic level upon innate immune cell activation is required for the secretion of IFN-γ and direct cytotoxicity. The glycolysis is increased by regulating the expression of citric acid and malic acid reverse transporter SLC25A1 and ACLY15 via SREBP signaling [74].

#### 3.2.6. B Cells CD19+

B cells are the lymphocyte component of adaptive immunity that produce immunoglobulins against familiar antigens. Their phenotypical activation in the adipose tissue promotes chemotaxis of neutrophils, T cells, and monocytes. These cells are somewhat unique because they use glycolysis and glutaminolysis in all resting and maturation states [75]. B cell accumulation generally precedes T cell accumulation in metabolic disorders, and promotes pro-inflammatory activation of T cells [68].

#### 3.2.7. T Cells CD3+ CD8+ (Cytotoxic)

These cells constitute a population of acquired immunity cells, and their abundance is increased in metabolically disadvantageous states. Energy requirements of these cells are maintained upon activation by glycolysis and glutaminolysis [70].

#### 3.2.8. T Cells CD3+ CD4+ (Helper)

T helper cells interact with the antigen-presenting cells like dendritic cells, macrophages, and B cells to modulate the inflammatory status of the target tissue. The maturation of these cells occurs in thymus and leads to amplification of several different subclasses of T cells that differ in their surface markers and cytokine profiles as summarized below [76].

Th1 cells are differentiated via pro-inflammatory IL-12 and IFN-γ signaling to release pro-inflammatory IFN-γ and TNF-α. Their bioenergetic profile depends on glycolysis and glutaminolysis upon activation.

Th2 cells are differentiated via IL-4 signaling to further secrete IL-4 (critical for survival of the B-type lymphocytes), IL-5, IL-10, and IL-13. Th2 and the majority of T helper cells described below maintain energy supply via glycolysis and partial preservation of oxidative phosphorylation.

Th9 cells are differentiated via IL-4 and TGF-β signaling to release IL-9.

Th17 cells are differentiated via IL-1β, IL-6, IL-23 and TGF-β signaling to release IL-17 in control of pathogenic infections and autoimmunity, as well as a series of IL-21/26 cytokines. Interestingly, high levels of glucose lead to the excessive differentiation and stimulation of Th17 cells and provoking inflammation in the host [77].

Th22 cells are differentiated via IL-6 and TNF-α signaling to release IL-22.

Treg (CD3+ CD4+ FOXP3+) regulatory cells are differentiated via IL-2 and TGF-β signaling to release IL-10 and TGF-β. The former maintains the immunosuppressive function of Tregs. The bioenergetic profile of T regs is similar to naïve T cells and is primarily supported by fatty acid oxidation and oxidative phosphorylation [76].

## 4. Influence of Essential Minerals on Immunological Outcomes

Nutrients exert their role in innate immunity and inflammation at two major checkpoints: (i) gut-associated lymphoid tissue in the intestinal tract, and (ii) immune cell crosstalk and signaling in the different host tissues. Inadequate nutritional states caused by malnutrition, unhealthy diet, disorders associated with loss, or inability, essential nutrients lead to nutritional deficiencies that can directly affect immune and inflammatory status of the body. The interactions among nutrients within foods and diets add additional level of complexity to the expected immune outcomes. This is especially evident at the level of macronutrients through existence of the nutrient-specific appetite systems for proteins, carbohydrates, and fats [78]. In many animals, the appetite prioritizes proteins for reproductive success and carbohydrates for longevity [79], and the overall food intake increases as nutrient concentrations fall in the food due to compensatory feeding for these macronutrients [80]. As such, total energy intakes are highest on diets with low protein and/or low carbohydrate content [81], and this unobvious discrepancy may form the basis for many lifestyle metabolic disorders.

While a similar seeking behavior may exist for at least two micronutrients, sodium and calcium, the remaining micronutrients are maintained within healthy limits by continuous intake from the available variety of foods. Essential minerals form a subgroup of these micronutrients with high implications for immune health. These include major—calcium (Ca), phosphorus (P), sodium (Na), potassium (K), magnesium (Mg), chloride (Cl), sulfur (S), and trace minerals—iron (Fe), zinc (Zn), copper (Cu), manganese (Mn), selenium (Se), iodine (I), molybdenum (Mo), and possibly trivalent chromium (Cr) and fluorine (F). Minerals play a vital role in maintaining integrity of various physiological and metabolic processes occurring within living tissues. Their regulatory effects on immune function have been defined to a certain extent, and inadequate levels of minerals have been reported to alter immune competence in humans [82].

A balanced and diverse diet can commonly support all essential minerals for the body [83], whether it is based on natural or fortified foods, yet modern lifestyles, dietary patterns, and agricultural production system has changed this equilibrium towards common inadequacies in some minerals (Table 1). The US data based on the National Health and Nutrition Examination Surveys (NHANES) supported prominent micronutrient inadequacies in the US population, with a particular focus on calcium, potassium, magnesium, and iron [84,85]. In Western diets, severe deficiencies occur only when minerals are not obtained in sufficient amounts or are not absorbed from the diet due to inaccessible chemical formulation or malabsorption disorders of the gastrointestinal tract, especially in patients on parenteral nutrition, elderly, and children with inborn errors of metabolism who require very specialized diets. This is in contrast to many developing countries where a variety of food choices is limited, the risk of economically driven malnutrition is high, and vegan diets are more widespread [86]. Individuals sustaining a vegetarian diet may require up to 50% more dietary minerals due to the plant phytates consumed. Inadequacies, however, are rather common all over the world, and the critical shortfall minerals with immune effects are described in more details below.

### 4.1. Calcium

Calcium is critically important for healthy bones and teeth, muscle contraction and relaxation, nerve functioning, blood clotting, blood pressure regulation, and the health of immune system. Calcium is tightly regulated and rarely varies from physiologic levels of 8.8–10.4 mg/dL serum (4.7–5.2 mg/dL if ionized) [87]. Calcium binds to bile acids and fatty acids to form insoluble complexes that protect the intestinal cells of the gut and maintain the immunological integrity of the intestine, in part by suppressing cell proliferation by promoting differentiation or apoptosis [88]. Calcium not only facilitates T-cell activation but also modulates the distinctive metabolic changes that arise in different T-cell subsets and developmental stages. The calcium influx is defective and the calcium efflux is increased in autophagy-deficient T cells, which leads to a decreased level of lymphocyte activation [89].

### 4.2. Potassium

Potassium is a systemic electrolyte that supports nerve transmission, muscle contraction, water balance, and energy production. Historically, potassium was used to treat symptoms of chronic cough as a mucus expectorant, and is expected to have similar effects at other mucosal surfaces, including gastric secretion and gastrointestinal tissue synthesis [90]. Normal serum potassium values are in 3.5–5 mmol/L range. Potassium balances sodium effects on innate immune system in part by inhibiting the NLRC4 inflammasome [91]. In the instances of insulin resistance, the body enters the catabolic state that suppresses the T lymphocyte-dependent adaptive immune system and drives inflammation in a continuous attempt to repair the damaged tissues, while unable to complete the immune sequence. Sodium/potassium pump is critical to ionic integrity of the lymphocyte under the conditions of insulin resistance and depleted potassium stores, thus preventing the lymphocyte to proceed in its cycle [92].

### 4.3. Magnesium

Magnesium is found in healthy bone tissue, this element also supports muscle contraction, nerve transmission, health of immune system, as well as cellular energy production and protein synthesis. Normal serum magnesium levels are between 1.8 and 2.2 mg/dL [93]. Magnesium acts as cofactors for enzymatic activation in multiple biochemical pathways such as glycolysis and the Krebs cycle. It is a co-factor for immunoglobulin synthesis, C′3 convertase, antibody-dependent cytolysis, immune cell adherence, IgM lymphocyte binding, macrophage response to lymphokines, and T or B cell adherence [94].

### 4.4. Iron

Iron is a critical part of red blood cell hemoglobin that ensures oxygen transfer and contributes to energy metabolism in all tissues as an electron transfer medium. Iron is also an integrated part of many enzyme systems that regulate cell regulation/proliferation, DNA synthesis, and electron transport in the mitochondria. Iron differs from other body minerals due to the absence of any physiological process of excretion. Normal value range is 60–170 μg/dL [95]. Iron is somewhat unique that it participates in nutritional immunity, an active withdrawal of iron from circulation in response to the infection [96]. Iron is pro-inflammatory both in macrophages and neutrophils when present in excess and not properly stored within ferritin, thus strengthening the elimination of pathogens at the expense of higher levels of tissue inflammation [97]. Iron also selectively promotes Th2 over Th1 cells differentiation and activity via INF-γ signaling, as well as contributes to the modulation of Treg due to the imbalance at the transferrin receptor CD71, an iron uptake protein [98]. The opposing effect is observed in B cells where iron promotes proliferation [99]. Downstream of the immune cell signaling, however, iron deficiency led to the downregulation of the antibody responses [100].

### 4.5. Other Major Minerals (Phosphorus, Sodium, Chloride, Sulfur)

Currently there are no critical shortcomings in this group of minerals as long as a balanced diet is maintained in the target population. Other than obvious participation in cell signaling and energy metabolism, the interactions between phosphorus and the immune system are inconsistent. Similarly, even though sodium can amplify inflammatory macrophage and T cell responses, translational evidence for the effects of dietary salt on human immunity is scarce [101]. Chloride, the most abundant anion in humans, is actively accumulated in the intracellular space of the myeloid cells such as neutrophils and macrophages, and reacts with hydrogen peroxide via phagolysosome hypochlorous acid to produce the defensive hypochlorous acid [102].

Sulfur is obtained from diet mostly in the form of sulfur amino acids and contributes to regulation of immune health via the metabolites such as glutathione, homocysteine, and taurine. Glutathione is the major storage form of sulfur in the body, as neither cysteine nor methionine are stored, and any excess is readily oxidized to sulfate and excreted in urine, often in the form of phase II metabolites of dietary pharmacophores [103]. Sulfur also contributes to immune health with production of tissue and colonic (microbiota) hydrogen sulfide that decreases the severity of various immune-mediated diseases [104], but can be detrimental in the pathogenesis of infectious diseases such as tuberculosis [105].

### 4.6. Other Trace Minerals (Zinc, Copper, Selenium, Manganese)

Trace mineral deficiency is not commonly seen in the developed regions unless associated with aging and chronic disorders. Patients with an acquired form of deficiency usually are unable to maintain intake, absorb, metabolize, or excrete the mineral efficiently. Precise assessment of the mineral status, however, is challenging because commonly used measurements in the clinic do not directly reflect mineral status in the target tissues where minerals tend to accumulate [106]. Even though healthy diets tend to cost more, it becomes increasingly evident that many nutritional inadequacies are driven by the consumer preference of low nutrient, high energy density diets at the same price point [107]. Paired with a dramatic decrease in consumption of organ meats that historically served as a good source of minerals for an omnivore population [108], selected trace mineral inadequacies may still be expected. Nutritional risks for trace mineral inadequacies include lack of meat intake, excess dietary phytates (legumes, seeds, whole grains) or oxalates (sorrel, spinach, okra, nuts, and tea). Impaired gastrointestinal absorption due to chronic gastrointestinal and metabolic diseases usually manifests itself by redistribution of body mineral stores away from the epithelium in the gut and skin, thus allowing for an increase of autoimmune disorders associated with these tissues [109].

Among the trace minerals discussed, zinc stands out in its requirement for several classes of catalytic enzymes such as matrix metalloproteinases, liver alcohol dehydrogenase, carbonic anhydrase, and transcriptional zinc finger proteins. Zinc is critical in reproductive health [110] and immune system, where it has a significant effect on the normal functioning of macrophages, neutrophils, natural killer cells, and complement activity, yet it cannot be stored in the body and must be replenished continuously [109]. Zinc inadequacy is a risk factor for the epithelial barrier integrity, both in the skin, gut (diarrhea), and lungs (viral infections). The immune effects are mediated in part by incorrect activation and maturation of T and B cells, and unbalanced ratio skewed in the direction of Th1 and Th17 pro-inflammatory phenotypes [111]. Increased recruitment of zinc into the activated immune cells and away from blood circulation and epithelial tissues may be essential to ensure transcription and translation of the acute phase proteins, but further depletes the available stores [112].

Copper is a cofactor for cytochrome c oxidase, the terminal enzyme in the electron transport chain that is critical for oxidative phosphorylation. This determines copper applicability to the proper functioning of organs and metabolic processes, stimulation of the immune system to fight infections, and repair of injured tissues [113]. Its relevance to immune system is mediated by iron and protein metabolism, as copper-containing ceruloplasmin is an important antioxidant component of ferroxidase, erythrocyte superoxide dismutase, and diamine oxidase [114]. Copper deficiencies are associated with impaired proliferation of T lymphocytes, decreased IL-2 production, and decreased activity of phagocytes, B-lymphocytes and natural killer cells [115]. In the presence of inflammation, plasma copper and ceruloplasmin concentrations are increased, resulting in increased protection against pathogens and oxygen radicals.

The relationship between the remaining trace minerals and immune function is less well documented in humans. Selenium is a part of the glutathione peroxidase and iodothyronine deiodinase enzyme systems, and plasma selenium levels correlate with the CD4+ counts and differentiation of CD4+ T-cells into Th1 cells [116]. Manganese is a co-factor of several proteins including superoxide dismutase [117]. Iodine (synthesis of thyroid hormones), molybdenum (mitochondrial bioenergetics), chromium (glucose and lipid metabolism), and fluorine (bone health) may have effects on immune health, but these are not well-defined. Of critical interest, both zinc and copper are classical examples of micronutrients that undergo spatiotemporal alterations in tissues during the onset and resolution of the inflammatory process [118]. Increased recruitment of zinc and copper into the immune cells at the time of infection or immune activation strengthens host defenses against pathogens by direct toxicity, as well as indirect increases in free radical formation and activation of the central enzymes in cellular metabolism [119]. The recently emerging evidence suggests that other trace elements such as selenium, manganese, or molybdenum may participate in similar processes and express similar transient increases in the activated immune cells [120].

## 5. Changes in the Mineral Content of Foods

Domestication of agricultural crops resulted in significant losses in biodiversity (crop package), complex carbohydrates, micronutrients, and dietary fiber in modern diets [20]. Limited ability to absorb, store, and metabolize these nutrients due to environmental or soil impacts, socioeconomic conditions, and health status may further lead to the development of multiple mineral inadequacies in the susceptible populations [121]. While food fortification strategies certainly help with most commonly identified deficiencies (iron, zinc, iodine), important opportunities for tackling multiple mineral malnutrition are missed by targeting single foods for single nutrients [21].

### 5.1. Agrociltural Food Production

Nutritional values of plant and animal foods changed during the selective domestication and breeding events all over the world. Energy density of everyday foods in the form of increased calorie retention has increased dramatically both in plants (accumulation of carbohydrates at the expense of protein and mineral content [122]) and animals (accumulation of saturated fat and cholesterol at the expense of unsaturated fat and minerals [123]).

Technological advances that allow for production of refined sugars and oils also increased high energy density of processed foods yet contributed negligible amounts of micronutrients to human diets. This transition formed a modern energy dense diet in which the total food energy is derived from 51.8% carbohydrate, 32.8% fat, and 15.4% protein [124]. Finally, nutritionary dense organ meats (offal) such as liver, heart, kidney, brain, tripe, intestine, and sweetbreads (thymus and pancreas) has fallen out of many modern diets [108]. These tissues usually contain higher amounts of minerals than skeletal muscles, as shown both for beef [125] and lamb [126].

Limited information is available to estimate contribution of different food groups to mineral intake at the populational level. For a few selected minerals such as phosphorus, potassium, magnesium, zinc, copper and manganese, the older data from British households suggested that total plant foods and fruits & vegetables contributed to daily diets 37% and 14% phosphorus, 60% and 45% potassium, 65% and 28% magnesium, 36% and 11% zinc, 61% and 24% copper, and 93% and 27% manganese, respectively [127]. These numbers, however, are significantly affected by systematic declines in the mineral content of fruits and vegetables as shown for the UK (sodium, calcium, magnesium, copper, and iron between 1940 and 2019), USA (calcium, phosphorus, copper and iron between 1950–2009), Finland (potassium, manganese, zinc and copper between 1970–2000s), Australia (iron and zinc between 1980–2000s) as summarized recently [21] (Table 2).

### 5.2. Bioavailability, Fortification, and Dietary Guidelines

A recent significant rise in alternative plant and cellular culture protein-based foods is another unknown in mineral health status of the populations that consume significant amounts of these products, since most minerals present in the whole animal and plant foods exist in biological complexes consisting of coenzymes and trace element activators.

This places additional constrains on absorption, fortification, and direct supplementation with micronutrients, especially among rapidly developing infants and children [121]. For these reasons, fortification of foods with micronutrients is often performed for iodine, iron, as well as vitamins A and D, and several B vitamins (thiamine B1, riboflavin B2, and niacin B3). Furthermore, increased access to nutritious foods, including products fortified with micronutrients (especially for iron, calcium, zinc and folate), supports dietary adequacy for most individuals in the developing populations [128]. However beneficial, micronutrient supplementation also comes with an increased health risk or no added health benefits for certain populations, as shown in clinical settings for countries with a high endemic burden of infectious diseases [129,130], or in several recent meta-analyses performed in industrialized countries [131,132].

Minerals in the food matrices are further bound to various organic and inorganic compounds (multiforms) which directly affect their solubility, absorption, and bioavailability. Food processing can have a substantial influence on the mineral bioavailability, both in the direction of irreversible loss or reduction, and more rarely in the direction of increased uptake [133]. Localization and activity of ion channels, transport proteins, epithelial integrity in the target areas of the gastrointestinal tract can also significantly modulate mineral uptake by the host. In instances when mineral chelation to phytates, polyphenols, tannins, lectins, dietary fibers, and proteins prevents their absorption in the upper intestine and enhances delivery of these minerals to the colonic lumen, gut microbiome may provide additional benefits through generation of short-chain fatty acids, reduced luminal pH (acidification), and increased mineral solubility [134]. Their assessment in human tissues other than biological liquids is challenging, as serum and urine mineral levels do not precisely reflect tissue micronutrient content [82] (Table 3).

Obtaining higher levels of minerals from diverse and whole food diets for a healthy individual without chronic gastrointestinal disorders is rather straightforward. The preferred dietary choices generally overlap with foods proposed to prevent and promote the recovery from depressive disorders [135], including bivalves (oysters, clams, or mussels), organ meats (liver, spleen, kidneys, or heart), and leafy greens (watercress, spinach, mustard, turnip, or Swiss chard). As a part of a healthy dietary pattern, this selection aligns well with the current guidelines on using nutrient-dense foods to meet daily nutrient requirements without consuming excessive calories [136].

## 6. Conclusions

Crucial functionally of minerals in application to metabolic and immune health cannot be overlooked. This is especially applicable to chronic metabolic and pro-inflammatory states that take time to develop and resolve. Recent developments in our understanding of absorption and bioavailability specific to each mineral, their abundance in circulation and target tissues represent a series of major advances in uncovering the nutritional basis of minerals intake and their application to human health. However, our understanding of the metabolically restrictive environments of the inflamed tissues, the critical dependence on aerobic glycolysis to supply immune cells with energy to perform immune functions, and mineral fluxes into the target tissues and back into circulation in support of these changes remain very fragmented.

The connection between the transitory and permanent states of insulin resistance associated with most metabolic and immune disorders is largely not explored, even though the knowledge about the connection between type I diabetes and an impaired immune response exists [137]. Redistribution of metabolic fluxes during the prolonged immune activation from pyruvate to lactate (outside of the TCA cycle towards NADH production and biosynthesis), glutamine to pyruvate (to compensate for the former) and citrate (for added synthesis of fatty acids and lipid species) leads to dysfunctional regulation of developmental metabolic programs of the immune cells. The future studies may discover that these metabolic states define a broader spectrum of cell subpopulations exemplified by M1 and M2 macrophage extremes, and mediated both by the inflammasome (IL-1β) and non-inflammasome (TNF-α) pathways, as well as a balance between pro-inflammatory palmitate (C16:0) and anti-inflammatory palmitoleate (C16:1n7) signaling [138]. It is interesting to note that inhibition of the aspartate-aminotransferase AST enzyme that shunts the fragmented TCA cycle is sufficient to promote mitochondrial respiration, inhibit nitric oxide and IL-6 production, and decrease M1 macrophage polarization [139].

Successful nutritional care of metabolic and immune outcomes with essential minerals is an important goal, as many mineral deficiencies and inadequacies are difficult to diagnose and quantify. Recent studies conducted with several minerals in the context of insulin resistance, systemic inflammation, and vaccination highlighted the need to further investigate these interventions in clinical settings [140], and emphasized the use of more effective multiforms for enhanced mineral delivery to the target tissues [106]. In the future, precise targeting of human mineral status and its contribution to overall health with interventions selected for desired physiological outcomes may be used to personalize nutrition strategies that help to manage chronical health disorders and promote optimal health.

## Figures and Tables

**Table 1:** Selected mineral intakes and inadequacies in the US population. Primary mineral inadequacies (shortfall micronutrients) are highlighted in red.

Minerals, mg	Mean Daily Intake from Naturally Occurring Foods, US Adults Ages ≥ 19 ^1^	Mean Daily Intake from All Foods Including Enriched and Fortified, US Population Ages ≥ 4 ^2^	RDA, Reference Daily Allowance for Ages ≥ 4 ^2^	EAR, % Less than Estimated Average Requirement ^2^
Calcium, Ca	856 mg	987 mg	1300 mg	−24.1%
Phosphorus, P	1308 mg	1350 mg	1250 mg	fair
Sodium, Na	3361 mg	3433 mg	1500 mg ^3^	excess
Potassium, K	2695 mg	2595 mg	4700 mg	−44.8%
Magnesium, Mg	278 mg	286 mg	420 mg	−31.9%
Iron, Fe	10.3 mg	15.1 mg	8–18 mg	−16.1%
Zinc, Zn	11.2 mg	11.7 mg	8–11 mg	fair
Copper, Cu	1.3 mg	1.3 mg	0.9 mg	fair
Selenium, Se	0.1 mg	0.1 mg	0.055 mg	fair
Manganese, Mn	n/a	2.1–4.1 mg	1.8–2.3 mg ^3^	fair

^1^ NHANES 2003–2006 [84]. ^2^ NHANES 2007–2010 [85]. ^3^ When RDA is not determined, AI (adequate daily intake) is listed instead. Major shortfall minerals are highlighted in red.

**Table 2:** Contributions by different groups of foods to intake of selected minerals in 1986 British households (mg per person per day) [127], as compared to mineral content of selected organ meats (lamb, per 100 g cooked edible portion) [126].

	Animal Foods				Refined		Plant Foods [127]					Lamb Organ Meats [126]						
Minerals, mg	Milk ^1^	Meat	Fish	Egg	Sug ^2^	Fats	Veg ^3^	Fruit	Bread	Flo ^4^	Cer ^5^	Int ^6^	Lung	Heart	Liver	Sto ^7^	Kid ^8^	Spleen
Calcium, Ca	- ^9^	-	-	-	-	-	-	-	-	-	-	18.6	8.9	5.1	5.0	52.7	9.4	7.6
Phosphorus, P	406	215	41	91	5	1	140	24	149	59	80	124	271	195	423	170	330	406
Sodium, Na	-	-	-	-	-	-	-	-	-	-	-	38	160	101	71	80	234	112
Potassium, K	529	305	61	62	3	9	893	194	175	81	98	75	298	261	315	155	310	409
Magnesium, Mg	44	21	5	5	1	1	46	14	46	12	22	21.9	22.2	29.0	28.3	25.3	30.6	30.8
Iron, Fe	-	-	-	-	-	-	-	-	-	-	-	1.4	8.4	3.8	6.1	4.9	4.4	22.8
Zinc, Zn	1.73	3.01	0.14	0.60	0.05	0.06	0.86	0.12	1.17	0.36	0.58	2.6	2.6	2.5	4.2	3.9	3.7	3.6
Copper, Cu	0.022	0.328	0.028	0.031	0.012	0.017	0.214	0.056	0.221	0.089	0.094	-	-	-	-	-	-	-
Selenium, Se	-	-	-	-	-	-	-	-	-	-	-	-	-	-	-	-	-	-
Manganese, Mn	0.03	0.10	0.01	0.01	0.01	0.01	0.54	0.16	1.06	0.26	0.36	0.01	0	0.04	0.27	0.19	0.05	0

^1^ Milk and milk products (dairy). ^2^ Refined sugars. ^3^ Vegetables, including potato. ^4^ Flour, cakes, and biscuits. ^5^ Other cereals and cereal products. ^6^ Intestines. ^7^ Stomach. ^8^ Kidney. ^9^ (-) Data not available.

**Table 3:** Absorption, transport, and normal value ranges of selected minerals.

Minerals	Absorption ^1^ Bioavailability	Ion Channels and Carrier Proteins	Main Transport Proteins	Normal Value Range (Serum) ^2^
Calcium, Ca	Duodenum 21–45%	TRPV6/5 influx NCX1/PMCA1b efflux	Albumin Calbindin	9.0–10.5 mg/dL 2.2–2.6 mmol/L
Phosphorus, P	Jejunum 40–70%	SLC34A1/2/3 influx Npt2 NaPi cotransporters	Free Albumin	2.8–4.5 mg/dL 0.97–1.45 mmol/L
Sodium, Na	Ileum/Colon 90%	NHE3 Na+/H+ exchange Na, K-ATPase efflux	Free	– 136–145 mmol/L
Potassium, K	Ileum/Colon 90%	Na, K-ATPase influx NKCC co-transporters	Free	– 3.5–5.1 mmol/L
Magnesium, Mg	Duodenum 40–60%	TRPM6/7 influx CNNM4/SLC41A1 efflux	Free Albumin	1.5–2.4 mg/dL 0.62–0.99 mmol/L
Iron, Fe	Duodenum 14–18%	HCP1/DMT1 influx FPN1 efflux	Ferritin Transferrin	50–150 μg/dL 9.2–27.5 μmol/L
Zinc, Zn	Jejunum 26–34%	SLC39A4/ZIP influx ZnT1/SLC30A1 efflux	Albumin	66–110 μg/dL 10.1–16.8 μmol/L
Copper, Cu	Jejunum 30–40%	SLC31A1/2 influx ATP7A efflux	Ceruloplasmin	7.0–15.5 μg/dL 11–24.2 μmol/L
Selenium, Se	Jejunum 69–86%	B(0)AT1/rBAT influx SLC26 family influx	Selenoproteins Selenosugars	11–16.5 μg/dL 0.6–1.5 μmol/L
Manganese, Mn	Jejunum 3–4%	DMT1/TRPM7 influx SLC30A10 efflux	Ceruloplasmin Albumin	30–90 ng/dL 5.5–16.4 nmol/L

^1^ Although many minerals are absorbed marginally along the entire length of the gastrointestinal tract, only major absorption areas are listed. Passive paracellular transport based on the large electrochemical gradients is not discussed. ^2^ Blood test normal values listed after 20th Edition Merck Manual of Diagnosis and Therapy (2018) that reflects medical practice and information in the US.

## Data Availability

Not applicable.

## References

[1] Wong M.C., McCarthy C., Fearnbach N., Yang S., Shepherd J., Heymsfield S.B. (2022). Emergence of the Obesity Epidemic: 6-Decade Visualization with Humanoid Avatars. Am. J. Clin. Nutr..

[2] Cantley J., Ashcroft F.M. (2015). Q&A: Insulin Secretion and Type 2 Diabetes: Why Do β-Cells Fail?. BMC Biol..

[3] O’Hearn M., Lauren B.N., Wong J.B., Kim D.D., Mozaffarian D. (2022). Trends and Disparities in Cardiometabolic Health Among U.S. Adults, 1999–2018. J. Am. Coll. Cardiol..

[4] MacKenna B., Kennedy N.A., Mehrkar A., Rowan A., Galloway J., Matthewman J., Mansfield K.E., Bechman K., Yates M., Brown J. (2022). Risk of Severe COVID-19 Outcomes Associated with Immune-Mediated Inflammatory Diseases and Immune-Modifying Therapies: A Nationwide Cohort Study in the OpenSAFELY Platform. Lancet Rheumatol..

[5] Boutari C., Mantzoros C.S. (2022). A 2022 Update on the Epidemiology of Obesity and a Call to Action: As Its Twin COVID-19 Pandemic Appears to Be Receding, the Obesity and Dysmetabolism Pandemic Continues to Rage On. Metabolism.

[6] Habbab R.M., Bhutta Z.A. (2020). Prevalence and Social Determinants of Overweight and Obesity in Adolescents in Saudi Arabia: A Systematic Review. Clin. Obes..

[7] Oh T.J., Lee H., Cho Y.M. (2022). East Asian Perspectives in Metabolic and Bariatric Surgery. J. Diabetes Investig..

[8] Calle M.C., Fernandez M.L. (2012). Inflammation and Type 2 Diabetes. Diabetes Metab..

[9] Rani V., Deep G., Singh R.K., Palle K., Yadav U.C.S. (2016). Oxidative Stress and Metabolic Disorders: Pathogenesis and Therapeutic Strategies. Life Sci..

[10] Tkachenko O., Polishchuk I., Gorchakova N., Zaychenko H. (2020). Metabolic Syndrome and Lipid Metabolism Disorders: Molecular and Biochemical Aspects. Acta Fac. Med. Naissensis.

[11] Andersen C.J., Murphy K.E., Fernandez M.L. (2016). Impact of Obesity and Metabolic Syndrome on Immunity12. Adv. Nutr..

[12] Ginhoux F., Guilliams M. (2016). Tissue-Resident Macrophage Ontogeny and Homeostasis. Immunity.

[13] Medzhitov R. (2008). Origin and Physiological Roles of Inflammation. Nature.

[14] De Fano M., Bartolini D., Tortoioli C., Vermigli C., Malara M., Galli F., Murdolo G. (2022). Adipose Tissue Plasticity in Response to Pathophysiological Cues: A Connecting Link between Obesity and Its Associated Comorbidities. Int. J. Mol. Sci..

[15] Fajgenbaum D.C., June C.H. (2020). Cytokine Storm. N. Engl. J. Med..

[16] Rathinasabapathy T., Sakthivel L.P., Komarnytsky S. (2022). Plant-Based Support of Respiratory Health during Viral Outbreaks. J. Agric. Food Chem..

[17] Soderholm A.T., Pedicord V.A. (2019). Intestinal Epithelial Cells: At the Interface of the Microbiota and Mucosal Immunity. Immunology.

[18] Mariathasan S., Weiss D.S., Newton K., McBride J., O’Rourke K., Roose-Girma M., Lee W.P., Weinrauch Y., Monack D.M., Dixit V.M. (2006). Cryopyrin Activates the Inflammasome in Response to Toxins and ATP. Nature.

[19] Arpaia N., Campbell C., Fan X., Dikiy S., van der Veeken J., deRoos P., Liu H., Cross J.R., Pfeffer K., Coffer P.J. (2013). Metabolites Produced by Commensal Bacteria Promote Peripheral Regulatory T-Cell Generation. Nature.

[20] Komarnytsky S., Retchin S., Vong C.I., Lila M.A. (2022). Gains and Losses of Agricultural Food Production: Implications for the Twenty-First Century. Annu. Rev. Food Sci. Technol..

[21] Mayer A.-M.B., Trenchard L., Rayns F. (2022). Historical Changes in the Mineral Content of Fruit and Vegetables in the UK from 1940 to 2019: A Concern for Human Nutrition and Agriculture. Int. J. Food Sci. Nutr..

[22] Rőszer T. (2015). Understanding the Mysterious M2 Macrophage through Activation Markers and Effector Mechanisms. Mediat. Inflamm..

[23] Murray P.J., Allen J.E., Biswas S.K., Fisher E.A., Gilroy D.W., Goerdt S., Gordon S., Hamilton J.A., Ivashkiv L.B., Lawrence T. (2014). Macrophage Activation and Polarization: Nomenclature and Experimental Guidelines. Immunity.

[24] Lawrence T., Gilroy D.W. (2007). Chronic Inflammation: A Failure of Resolution?. Int. J. Exp. Pathol..

[25] Nijhuis J., Rensen S.S., Slaats Y., van Dielen F.M.H., Buurman W.A., Greve J.W.M. (2009). Neutrophil Activation in Morbid Obesity, Chronic Activation of Acute Inflammation. Obes. (Silver Spring).

[26] Hotamisligil G.S. (2006). Inflammation and Metabolic Disorders. Nature.

[27] Dehghan M., Mente A., Zhang X., Swaminathan S., Li W., Mohan V., Iqbal R., Kumar R., Wentzel-Viljoen E., Rosengren A. (2017). Associations of Fats and Carbohydrate Intake with Cardiovascular Disease and Mortality in 18 Countries from Five Continents (PURE): A Prospective Cohort Study. Lancet.

[28] Le Couteur D.G., Solon-Biet S., Wahl D., Cogger V.C., Willcox B.J., Willcox D.C., Raubenheimer D., Simpson S.J. (2016). New Horizons: Dietary Protein, Ageing and the Okinawan Ratio. Age Ageing.

[29] Wali J.A., Raubenheimer D., Senior A.M., Le Couteur D.G., Simpson S.J. (2021). Cardio-Metabolic Consequences of Dietary Carbohydrates: Reconciling Contradictions Using Nutritional Geometry. Cardiovasc. Res..

[30] Kubota N., Kubota T., Kajiwara E., Iwamura T., Kumagai H., Watanabe T., Inoue M., Takamoto I., Sasako T., Kumagai K. (2016). Differential Hepatic Distribution of Insulin Receptor Substrates Causes Selective Insulin Resistance in Diabetes and Obesity. Nat. Commun..

[31] Metz H.E., Kargl J., Busch S.E., Kim K.-H., Kurland B.F., Abberbock S.R., Randolph-Habecker J., Knoblaugh S.E., Kolls J.K., White M.F. (2016). Insulin Receptor Substrate-1 Deficiency Drives a Proinflammatory Phenotype in KRAS Mutant Lung Adenocarcinoma. Proc. Natl. Acad. Sci. USA.

[32] Chen G., Goeddel D.V. (2002). TNF-R1 Signaling: A Beautiful Pathway. Science.

[33] Weyer C., Yudkin J.S., Stehouwer C.D.A., Schalkwijk C.G., Pratley R.E., Tataranni P.A. (2002). Humoral Markers of Inflammation and Endothelial Dysfunction in Relation to Adiposity and in Vivo Insulin Action in Pima Indians. Atherosclerosis.

[34] McLaughlin T., Ackerman S.E., Shen L., Engleman E. (2017). Role of Innate and Adaptive Immunity in Obesity-Associated Metabolic Disease. J. Clin. Investig..

[35] Shao B.-Z., Xu Z.-Q., Han B.-Z., Su D.-F., Liu C. (2015). NLRP3 Inflammasome and Its Inhibitors: A Review. Front. Pharmacol..

[36] Tencerova M., Aouadi M., Vangala P., Nicoloro S.M., Yawe J.C., Cohen J.L., Shen Y., Garcia-Menendez L., Pedersen D.J., Gallagher-Dorval K. (2015). Activated Kupffer Cells Inhibit Insulin Sensitivity in Obese Mice. FASEB J..

[37] Fink L.N., Oberbach A., Costford S.R., Chan K.L., Sams A., Blüher M., Klip A. (2013). Expression of Anti-Inflammatory Macrophage Genes within Skeletal Muscle Correlates with Insulin Sensitivity in Human Obesity and Type 2 Diabetes. Diabetologia.

[38] Zhou T., Hu Z., Yang S., Sun L., Yu Z., Wang G. (2018). Role of Adaptive and Innate Immunity in Type 2 Diabetes Mellitus. J. Diabetes Res..

[39] Martin M.A., Goya L., Ramos S. (2017). Protective Effects of Tea, Red Wine and Cocoa in Diabetes. Evidences from Human Studies. Food Chem. Toxicol..

[40] Forbes J.M., Cooper M.E. (2013). Mechanisms of Diabetic Complications. Physiol. Rev..

[41] Duncan B.B., Schmidt M.I., Pankow J.S., Ballantyne C.M., Couper D., Vigo A., Hoogeveen R., Folsom A.R., Heiss G. (2003). Atherosclerosis Risk in Communities Study Low-Grade Systemic Inflammation and the Development of Type 2 Diabetes: The Atherosclerosis Risk in Communities Study. Diabetes.

[42] Aguirre V., Uchida T., Yenush L., Davis R., White M.F. (2000). The C-Jun NH(2)-Terminal Kinase Promotes Insulin Resistance during Association with Insulin Receptor Substrate-1 and Phosphorylation of Ser(307). J. Biol. Chem..

[43] Magkos F., Fraterrigo G., Yoshino J., Luecking C., Kirbach K., Kelly S.C., de Las Fuentes L., He S., Okunade A.L., Patterson B.W. (2016). Effects of Moderate and Subsequent Progressive Weight Loss on Metabolic Function and Adipose Tissue Biology in Humans with Obesity. Cell Metab..

[44] Williamson R.T. (1901). On the Treatment of Glycosuria and Diabetes Mellitus with Sodium Salicylate. Br. Med. J..

[45] Yuan M., Konstantopoulos N., Lee J., Hansen L., Li Z.W., Karin M., Shoelson S.E. (2001). Reversal of Obesity- and Diet-Induced Insulin Resistance with Salicylates or Targeted Disruption of Ikkbeta. Science.

[46] Hundal R.S., Petersen K.F., Mayerson A.B., Randhawa P.S., Inzucchi S., Shoelson S.E., Shulman G.I. (2002). Mechanism by Which High-Dose Aspirin Improves Glucose Metabolism in Type 2 Diabetes. J. Clin. Investig..

[47] Zatterale F., Longo M., Naderi J., Raciti G.A., Desiderio A., Miele C., Beguinot F. (2020). Chronic Adipose Tissue Inflammation Linking Obesity to Insulin Resistance and Type 2 Diabetes. Front. Physiol..

[48] Man A.W.C., Zhou Y., Xia N., Li H. (2020). Involvement of Gut Microbiota, Microbial Metabolites and Interaction with Polyphenol in Host Immunometabolism. Nutrients.

[49] Grondin J.A., Kwon Y.H., Far P.M., Haq S., Khan W.I. (2020). Mucins in Intestinal Mucosal Defense and Inflammation: Learning From Clinical and Experimental Studies. Front. Immunol..

[50] Lee B., Moon K.M., Kim C.Y. (2018). Tight Junction in the Intestinal Epithelium: Its Association with Diseases and Regulation by Phytochemicals. J. Immunol. Res..

[51] Hase K., Kawano K., Nochi T., Pontes G.S., Fukuda S., Ebisawa M., Kadokura K., Tobe T., Fujimura Y., Kawano S. (2009). Uptake through Glycoprotein 2 of FimH(+) Bacteria by M Cells Initiates Mucosal Immune Response. Nature.

[52] Cornes J.S. (1965). Number, Size, and Distribution of Peyer’s Patches in the Human Small Intestine: Part I The Development of Peyer’s Patches. Gut.

[53] Rooks M.G., Garrett W.S. (2016). Gut Microbiota, Metabolites and Host Immunity. Nat. Rev. Immunol..

[54] Palatini K.M., Durand P.-J., Rathinasabapathy T., Esposito D., Komarnytsky S. (2016). Bitter Receptors and Glucose Transporters Interact to Control Carbohydrate and Immune Responses in the Gut. FASEB J..

[55] Nestares T., Martín-Masot R., Labella A., Aparicio V.A., Flor-Alemany M., López-Frías M., Maldonado J. (2020). Is a Gluten-Free Diet Enough to Maintain Correct Micronutrients Status in Young Patients with Celiac Disease?. Nutrients.

[56] Naik A., Venu N. (2012). Nutrition Care in Adult Inflammatory Bowel Disease. Pract. Gastroenterol..

[57] Couper C., Doriot A., Siddiqui M.T.R., Steiger E. (2021). Nutrition Management of the High-Output Fistulae. Nutr. Clin. Pr..

[58] Holmberg F.E.O., Pedersen J., Jørgensen P., Soendergaard C., Jensen K.B., Nielsen O.H. (2018). Intestinal Barrier Integrity and Inflammatory Bowel Disease: Stem Cell-Based Approaches to Regenerate the Barrier. J. Tissue Eng. Regen Med..

[59] Xia L., Oyang L., Lin J., Tan S., Han Y., Wu N., Yi P., Tang L., Pan Q., Rao S. (2021). The Cancer Metabolic Reprogramming and Immune Response. Mol. Cancer.

[60] Pearce E.L., Pearce E.J. (2013). Metabolic Pathways in Immune Cell Activation and Quiescence. Immunity.

[61] Nielsen S.T., Janum S., Krogh-Madsen R., Solomon T.P., Møller K. (2015). The Incretin Effect in Critically Ill Patients: A Case—Control Study. Crit. Care.

[62] Berlanga-Acosta J., Mendoza-Marí Y., Rodríguez-Rodríguez N., García del Barco Herrera D., García-Ojalvo A., Fernández-Mayola M., Guillén-Nieto G., Valdés-Sosa P.A. (2020). Burn Injury Insulin Resistance and Central Nervous System Complications: A Review. Burn. Open.

[63] Sonagra A.D., Biradar S.M., Dattatreya K., Jayaprakash Murthy D.S. (2014). Normal Pregnancy—A State of Insulin Resistance. J. Clin. Diagn Res..

[64] Mukhopadhyay R., Jia J., Arif A., Ray P.S., Fox P.L. (2009). The GAIT System: A Gatekeeper of Inflammatory Gene Expression. Trends Biochem. Sci..

[65] Tannahill G.M., Curtis A.M., Adamik J., Palsson-McDermott E.M., McGettrick A.F., Goel G., Frezza C., Bernard N.J., Kelly B., Foley N.H. (2013). Succinate Is an Inflammatory Signal That Induces IL-1β through HIF-1α. Nature.

[66] Zhang X., Zink F., Hezel F., Vogt J., Wachter U., Wepler M., Loconte M., Kranz C., Hellmann A., Mizaikoff B. (2020). Metabolic Substrate Utilization in Stress-Induced Immune Cells. Intensive Care Med. Exp..

[67] Mendoza R.P., Fudge D.H., Brown J.M. (2021). Cellular Energetics of Mast Cell Development and Activation. Cells.

[68] Lee Y.S., Wollam J., Olefsky J.M. (2018). An Integrated View of Immunometabolism. Cell.

[69] Reilly S.M., Saltiel A.R. (2017). Adapting to Obesity with Adipose Tissue Inflammation. Nat. Rev. Endocrinol..

[70] Castoldi A., Naffah de Souza C., Câmara N.O.S., Moraes-Vieira P.M. (2016). The Macrophage Switch in Obesity Development. Front. Immunol..

[71] O’Neill L.A.J. (2015). A Broken Krebs Cycle in Macrophages. Immunity.

[72] Zigmond E., Bernshtein B., Friedlander G., Walker C.R., Yona S., Kim K.-W., Brenner O., Krauthgamer R., Varol C., Müller W. (2014). Macrophage-Restricted Interleukin-10 Receptor Deficiency, but Not IL-10 Deficiency, Causes Severe Spontaneous Colitis. Immunity.

[73] Stefanovic-Racic M., Yang X., Turner M.M.S., Mantell B.S., Stolz D.B., Sumpter T.L., Sipula I.J., Dedousis N., Scott D.K., Morel P.A. (2012). Dendritic Cells Promote Macrophage Infiltration and Comprise a Substantial Proportion of Obesity-Associated Increases in CD11c+ Cells in Adipose Tissue and Liver. Diabetes.

[74] Assmann N., O’Brien K.L., Donnelly R.P., Dyck L., Zaiatz-Bittencourt V., Loftus R.M., Heinrich P., Oefner P.J., Lynch L., Gardiner C.M. (2017). Srebp-Controlled Glucose Metabolism Is Essential for NK Cell Functional Responses. Nat. Immunol..

[75] Kane H., Lynch L. (2019). Innate Immune Control of Adipose Tissue Homeostasis. Trends Immunol..

[76] Golubovskaya V., Wu L. (2016). Different Subsets of T Cells, Memory, Effector Functions, and CAR-T Immunotherapy. Cancers.

[77] Zhang D., Jin W., Wu R., Li J., Park S.-A., Tu E., Zanvit P., Xu J., Liu O., Cain A. (2019). High Glucose Intake Exacerbates Autoimmunity through Reactive-Oxygen-Species-Mediated TGF-β Cytokine Activation. Immunity.

[78] Simpson S.J., Raubenheimer D. (2014). Perspective: Tricks of the Trade. Nature.

[79] Solon-Biet S.M., McMahon A.C., Ballard J.W.O., Ruohonen K., Wu L.E., Cogger V.C., Warren A., Huang X., Pichaud N., Melvin R.G. (2014). The Ratio of Macronutrients, Not Caloric Intake, Dictates Cardiometabolic Health, Aging, and Longevity in Ad Libitum-Fed Mice. Cell Metab..

[80] Sørensen A., Mayntz D., Raubenheimer D., Simpson S.J. (2008). Protein-Leverage in Mice: The Geometry of Macronutrient Balancing and Consequences for Fat Deposition. Obes. (Silver Spring).

[81] Simpson S.J., Le Couteur D.G., Raubenheimer D. (2015). Putting the Balance Back in Diet. Cell.

[82] Gharibzahedi S.M.T., Jafari S.M. (2017). The Importance of Minerals in Human Nutrition: Bioavailability, Food Fortification, Processing Effects and Nanoencapsulation. Trends Food Sci. Technol..

[83] Lukaski H.C. (2004). Vitamin and Mineral Status: Effects on Physical Performance. Nutrition.

[84] Fulgoni V.L., Keast D.R., Bailey R.L., Dwyer J. (2011). Foods, Fortificants, and Supplements: Where Do Americans Get Their Nutrients?. J. Nutr..

[85] Wallace T.C., McBurney M., Fulgoni V.L. (2014). Multivitamin/Mineral Supplement Contribution to Micronutrient Intakes in the United States, 2007–2010. J. Am. Coll. Nutr..

[86] Drake V. Micronutrient Inadequacies in the US Population: An Overview 2017. https://lpi.oregonstate.edu/mic/micronutrient-inadequacies/overview.

[87] Zheng J., Zeng X., Wang S. (2015). Calcium Ion as Cellular Messenger. Sci. China Life Sci..

[88] Milner J.A., McDonald S.S., Anderson D.E., Greenwald P. (2001). Molecular Targets for Nutrients Involved with Cancer Prevention. Nutr. Cancer.

[89] Jia W., He M., He Y.-W., Mcleod I. (2013). Autophagy, a Novel Pathway to Regulate Calcium Mobilization in T Lymphocytes. Front. Immunol..

[90] Khalili H., Malik S., Ananthakrishnan A.N., Garber J.J., Higuchi L.M., Joshi A., Peloquin J., Richter J.M., Stewart K.O., Curhan G.C. (2016). Identification and Characterization of a Novel Association between Dietary Potassium and Risk of Crohn’s Disease and Ulcerative Colitis. Front. Immunol..

[91] Arlehamn C.S.L., Pétrilli V., Gross O., Tschopp J., Evans T.J. (2010). The Role of Potassium in Inflammasome Activation by Bacteria. J. Biol. Chem..

[92] Hill A.F., Polvino W.J., Wilson D.B. (2005). The Significance of Glucose, Insulin and Potassium for Immunology and Oncology: A New Model of Immunity. J. Immune Based Vaccines.

[93] Swaminathan R. (2003). Magnesium Metabolism and Its Disorders. Clin. Biochem. Rev..

[94] Tam M., Gómez S., González-Gross M., Marcos A. (2003). Possible Roles of Magnesium on the Immune System. Eur. J. Clin. Nutr..

[95] von Drygalski A., Adamson J.W. (2013). Iron Metabolism in Man. JPEN J. Parenter Enter. Nutr..

[96] Cassat J.E., Skaar E.P. (2013). Iron in Infection and Immunity. Cell Host Microbe.

[97] Torti S.V., Torti F.M. (2020). Iron: The Cancer Connection. Mol. Asp. Med..

[98] Heintzman D.R., Fisher E.L., Rathmell J.C. (2022). Microenvironmental Influences on T Cell Immunity in Cancer and Inflammation. Cell Mol. Immunol..

[99] Ni S., Yuan Y., Kuang Y., Li X. (2022). Iron Metabolism and Immune Regulation. Front. Immunol..

[100] Jiang Y., Li C., Wu Q., An P., Huang L., Wang J., Chen C., Chen X., Zhang F., Ma L. (2019). Iron-Dependent Histone 3 Lysine 9 Demethylation Controls B Cell Proliferation and Humoral Immune Responses. Nat. Commun..

[101] Jobin K., Müller D.N., Jantsch J., Kurts C. (2021). Sodium and Its Manifold Impact on Our Immune System. Trends Immunol..

[102] Wang G. (2016). Chloride Flux in Phagocytes. Immunol. Rev..

[103] Nimni M.E., Han B., Cordoba F. (2007). Are We Getting Enough Sulfur in Our Diet?. Nutr. Metab..

[104] Dilek N., Papapetropoulos A., Toliver-Kinsky T., Szabo C. (2020). Hydrogen Sulfide: An Endogenous Regulator of the Immune System. Pharmacol. Res..

[105] Rahman M.A., Cumming B.M., Addicott K.W., Pacl H.T., Russell S.L., Nargan K., Naidoo T., Ramdial P.K., Adamson J.H., Wang R. (2020). Hydrogen Sulfide Dysregulates the Immune Response by Suppressing Central Carbon Metabolism to Promote Tuberculosis. Proc. Natl. Acad. Sci. USA.

[106] El-Khodor B.F., James K., Chang Q., Zhang W., Loiselle Y.R., Panda C., Hanania T. (2021). Elevation of Brain Magnesium with Swiss Chard and Buckwheat Extracts in an Animal Model of Reduced Magnesium Dietary Intake. Nutr. Neurosci..

[107] Visser M., Van Zyl T., Hanekom S.M., Baumgartner J., Van der Hoeven M., Taljaard-Krugell C., Smuts C.M., Faber M. (2021). Nutrient Density, but Not Cost of Diet, Is Associated with Anemia and Iron Deficiency in School-Age Children in South Africa. Nutrition.

[108] Hopkins H.T., Murphy E.W., Smith D.P. (1961). Minerals and Proximate Composition of Organ Meats. J. Am. Diet. Assoc..

[109] Sanna A., Firinu D., Zavattari P., Valera P. (2018). Zinc Status and Autoimmunity: A Systematic Review and Meta-Analysis. Nutrients.

[110] Fallah A., Mohammad-Hasani A., Colagar A.H. (2018). Zinc Is an Essential Element for Male Fertility: A Review of Zn Roles in Men’s Health, Germination, Sperm Quality, and Fertilization. J. Reprod. Infertil..

[111] Haase H., Rink L. (2014). Multiple Impacts of Zinc on Immune Function. Metallomics.

[112] Bonaventura P., Lamboux A., Albarède F., Miossec P. (2016). A Feedback Loop between Inflammation and Zn Uptake. PLoS ONE.

[113] Collins J.F., Klevay L.M. (2011). Copper. Adv. Nutr..

[114] Bost M., Houdart S., Oberli M., Kalonji E., Huneau J.-F., Margaritis I. (2016). Dietary Copper and Human Health: Current Evidence and Unresolved Issues. J. Trace Elem. Med. Biol..

[115] Tapiero H., Townsend D.M., Tew K.D. (2003). Trace Elements in Human Physiology and Pathology. Copper. Biomed. Pharm..

[116] Kieliszek M., Błażejak S. (2016). Current Knowledge on the Importance of Selenium in Food for Living Organisms: A Review. Molecules.

[117] Aschner M., Erikson K. (2017). Manganese. Adv. Nutr..

[118] Skaar E.P., Raffatellu M. (2015). Metals in Infectious Diseases and Nutritional Immunity. Metallomics.

[119] Weiss G., Carver P.L. (2018). Role of Divalent Metals in Infectious Disease Susceptibility and Outcome. Clin. Microbiol. Infect..

[120] Cunrath O., Bumann D. (2019). Host Resistance Factor SLC11A1 Restricts Salmonella Growth through Magnesium Deprivation. Science.

[121] Tako E. (2019). Dietary Trace Minerals. Nutrients.

[122] Harlan J.R., de Wet J.M.J., Price E.G. (1973). Comparative Evolution of Cereals. Evolution.

[123] Cordain L., Watkins B.A., Florant G.L., Kelher M., Rogers L., Li Y. (2002). Fatty Acid Analysis of Wild Ruminant Tissues: Evolutionary Implications for Reducing Diet-Related Chronic Disease. Eur. J. Clin. Nutr..

[124] Cordain L., Eaton S.B., Sebastian A., Mann N., Lindeberg S., Watkins B.A., O’Keefe J.H., Brand-Miller J. (2005). Origins and Evolution of the Western Diet: Health Implications for the 21st Century. Am. J. Clin. Nutr..

[125] van Heerden S.M., Morey L. (2014). Nutrient Content of South African C2 Beef Offal. Food Meas..

[126] Bester M., Schönfeldt H.C., Pretorius B., Hall N. (2018). The Nutrient Content of Selected South African Lamb and Mutton Organ Meats (Offal). Food Chem..

[127] Lewis J., Buss D.H. (1988). Trace Nutrients. 5. Minerals and Vitamins in the British Household Food Supply. Br. J. Nutr..

[128] Ferguson E.L., Watson L., Berger J., Chea M., Chittchang U., Fahmida U., Khov K., Kounnavong S., Le B.M., Rojroongwasinkul N. (2019). Realistic Food-Based Approaches Alone May Not Ensure Dietary Adequacy for Women and Young Children in South-East Asia. Matern Child. Health J..

[129] Sazawal S., Black R.E., Ramsan M., Chwaya H.M., Stoltzfus R.J., Dutta A., Dhingra U., Kabole I., Deb S., Othman M.K. (2006). Effects of Routine Prophylactic Supplementation with Iron and Folic Acid on Admission to Hospital and Mortality in Preschool Children in a High Malaria Transmission Setting: Community-Based, Randomised, Placebo-Controlled Trial. Lancet.

[130] Soofi S., Cousens S., Iqbal S.P., Akhund T., Khan J., Ahmed I., Zaidi A.K.M., Bhutta Z.A. (2013). Effect of Provision of Daily Zinc and Iron with Several Micronutrients on Growth and Morbidity among Young Children in Pakistan: A Cluster-Randomised Trial. Lancet.

[131] Mangione C.M., Barry M.J., Nicholson W.K., Cabana M., Chelmow D., Coker T.R., Davis E.M., Donahue K.E., Doubeni C.A., US Preventive Services Task Force (2022). Vitamin, Mineral, and Multivitamin Supplementation to Prevent Cardiovascular Disease and Cancer: US Preventive Services Task Force Recommendation Statement. JAMA.

[132] O’Connor E.A., Evans C.V., Ivlev I., Rushkin M.C., Thomas R.G., Martin A., Lin J.S. (2022). Vitamin and Mineral Supplements for the Primary Prevention of Cardiovascular Disease and Cancer: Updated Evidence Report and Systematic Review for the US Preventive Services Task Force. JAMA.

[133] Gibson R.S., Perlas L., Hotz C. (2006). Improving the Bioavailability of Nutrients in Plant Foods at the Household Level. Proc. Nutr. Soc..

[134] Bielik V., Kolisek M. (2021). Bioaccessibility and Bioavailability of Minerals in Relation to a Healthy Gut Microbiome. Int. J. Mol. Sci..

[135] LaChance L.R., Ramsey D. (2018). Antidepressant Foods: An Evidence-Based Nutrient Profiling System for Depression. World J. Psychiatry.

[136] DeSalvo K.B., Olson R., Casavale K.O. (2016). Dietary Guidelines for Americans. JAMA.

[137] Patry M., Teinturier R., Goehrig D., Zetu C., Ripoche D., Kim I.-S., Bertolino P., Hennino A. (2015). Βig-H3 Represses T-Cell Activation in Type 1 Diabetes. Diabetes.

[138] Talbot N.A., Wheeler-Jones C.P., Cleasby M.E. (2014). Palmitoleic Acid Prevents Palmitic Acid-Induced Macrophage Activation and Consequent P38 MAPK-Mediated Skeletal Muscle Insulin Resistance. Mol. Cell. Endocrinol..

[139] Jha A.K., Huang S.C.-C., Sergushichev A., Lampropoulou V., Ivanova Y., Loginicheva E., Chmielewski K., Stewart K.M., Ashall J., Everts B. (2015). Network Integration of Parallel Metabolic and Transcriptional Data Reveals Metabolic Modules That Regulate Macrophage Polarization. Immunity.

[140] Tourkochristou E., Triantos C., Mouzaki A. (2021). The Influence of Nutritional Factors on Immunological Outcomes. Front. Immunol..

